# Biorefining lignocellulose into feed and food: the case of sugarcane and a technology outlook

**DOI:** 10.3389/fbioe.2025.1653367

**Published:** 2025-08-14

**Authors:** Fernando Roberto Paz Cedeno, Gabriel P. Petrielli, Sergio R. de Medeiros, Alexandre Berndt, Thayse A. D. Hernandes, Antonio Bonomi, Carlos Driemeier

**Affiliations:** ^1^ Brazilian Biorenewables National Laboratory (LNBR), Brazilian Center for Research in Energy and Materials (CNPEM), Campinas, Brazil; ^2^ Embrapa Pecuária Sudeste, São Carlos, Brazil; ^3^ School of Mechanical Engineering, University of Campinas, Campinas, Brazil

**Keywords:** lignocellulose, bagasse, pretreatment, biorefinery, feed, protein

## Abstract

This Perspective explores how new technologies can expand lignocellulose biorefineries to include coproducts for animal feed and microbial protein with potential applications in human food. Using the Brazilian sugarcane industry as a case study, the analysis highlights synergies from the spatial coexistence of sugarcane and livestock, as well as economies of scale and product multiplicity in biorefineries. The technology outlook examines selected biomass pretreatments that can generate pretreated biomass with dual use: reactive intermediate for cellulosic ethanol production and ruminant nutrition. However, reliance on biorefined feed requires rebalancing ruminant diets and enhancing nutritional value. Emerging technologies for microbial protein production from biorefining streams are briefly contextualized, considering the potential addition in the diets of livestock and humans. In conclusion, research and development in this domain can unlock key opportunities to enhance the sustainability of bio-based value chains.

## 1 Introduction

Biomass is a valuable renewable carbon source with competing end uses for food, feed, materials, chemicals, fuels, and carbon removal (Muscat et al., 2020; Dees et al., 2023). Biorefineries fractionate and transform biomass components into a portfolio of renewable bio-based products. As elements of industrialization, biorefineries enhance the economic, societal, and environmental value obtained from biomass, partly mitigating the competition among the distinct biomass uses (Kumar et al., 2020; Paone et al., 2020; Singh et al., 2022).

Ethanol is the predominant biofuel globally, with production exceeding 100 Mm^3^ yr^-1^. Ethanol from cereal grain, chiefly corn, is obtained from biorefineries that coproduce edible oil and protein-rich distiller’s grains, widely used as feed (Scholey et al., 2016; Eckert et al., 2018). In the case of sugarcane, most industrial plants transform the sucrose-rich juice into both ethanol and edible sugar (de Souza Dias et al., 2015; Klein et al., 2019; Vandenberghe et al., 2022), further evidencing that coproduction of fuel, feed, and food is mainstream practice in first-generation (1G) ethanol technologies.

Biorefining of lignocellulose represents a technology frontier, often called second-generation (2G) technologies. Lignocellulose comprises the structural, non-edible fractions of plants, primarily cellulose, hemicelluloses, and lignin. As the most abundant type of biomass, lignocellulose serves as the main renewable carbon source (Langholtz, 2024). The sugarcane industry is currently leading the deployment of new lignocellulose biorefining technologies in commercial-scale cellulosic ethanol facilities based on bagasse and straw pretreatment, enzymatic hydrolysis, and sugar fermentation (Vandenberghe et al., 2022; Menezes et al., 2023). This leadership stems from several favorable conditions: the on-site availability of sugarcane bagasse (the fibrous residue left after juice extraction from the stalks); the additional availability of straw (leafy matter); and the synergies in integrating 2G technologies with the established 1G industry (Junqueira et al., 2017; Negrão et al., 2021; Nascimento et al., 2024).

In considering these 1G and 2G scenarios, it is sensible to evaluate the potential of expanding lignocellulose biorefining technology to include feed and food coproducts. This objective aligns with the bioenergy-livestock integration (BLI) studies in Brazil, which aim to identify opportunities for maximizing synergies and minimizing trade-offs in the joint production of food, feed, and bioenergy (Rinke Dias de Souza et al., 2021). Nonetheless, the BLI studies have not yet assessed the potential of lignocellulose biorefining to enhance an integrated food-feed-bioenergy system. Additionally, a comprehensive overview of lignocellulose biorefining into feed and food, beyond the specificities of the Brazilian context and sugarcane cases, is missing and could significantly contribute to the advancement of biorefining.

This Perspective addresses these knowledge gaps through a spatial analysis of the Brazilian Center-South (Section 2), a description of economies of scale and process streams in biorefineries (Section 3), a presentation of biomass pretreatments for multiproduct biorefining (Section 4), an explanation on ruminant diet rebalancing for biorefined feed (Section 5), and a contextualization on microbial protein integration into biorefinery products (Section 6). The concluding remarks (Section 7) emphasize the need for R&D to turn the identified potential into reality.

## 2 Sugarcane and livestock in Brazilian center-south

The potential geographic area for the integration between sugarcane and livestock productions was assessed in six states (GO, MT, MS, MG, PR, and SP) of the Brazilian Center-South region. These states collectively represent more than 90% of the country’s sugarcane area (CONAB, 2023) and around 63% of the country’s livestock (IBGE, 2022). Two scenarios of sugarcane production were considered: (*i*) the current cultivated area from the Canasat Project (Rudorff et al., 2010) and (*ii*) an expansion of sugarcane cultivation over pastureland inside the Sugarcane Agroecological Zoning (Manzatto et al., 2009) as a conservative approach to avoid cropland conversion (Hernandes et al., 2021). The availability of sugarcane biomass for both scenarios was estimated using the Crop Assessment Tool (CAT), quantified in tonnes of sugarcane (t_c_) on a wet stalk basis. The CAT model uses georeferenced climate data (Xavier et al., 2016) and provides attainable yields constrained by water availability. This model’s outputs align closely with commercial observations (Petrielli et al., 2023) and ensure better spatial resolution, replicability, and consistency across both current and expansion cultivation areas. Livestock production was extracted at the municipality level from Municipal Livestock Production for 2020 (IBGE, 2022). The comparison between sugarcane and livestock productions was performed at a geographic level of microregion to be consistent with the supply of an optimized sugarcane biorefinery with a processing capacity of at least 4 Mt_c_ yr^-1^ (Junqueira et al., 2016). For each microregion, the ratio between sugarcane biomass availability (t_c_) and livestock production in animal units (AU) was calculated (t_c_ AU^−1^) to examine the spatial coexistence of the two activities. AU is defined as one adult bovine equivalent weighing approximately 450 kg (IBGE, 2022).

**FIGURE 1:**
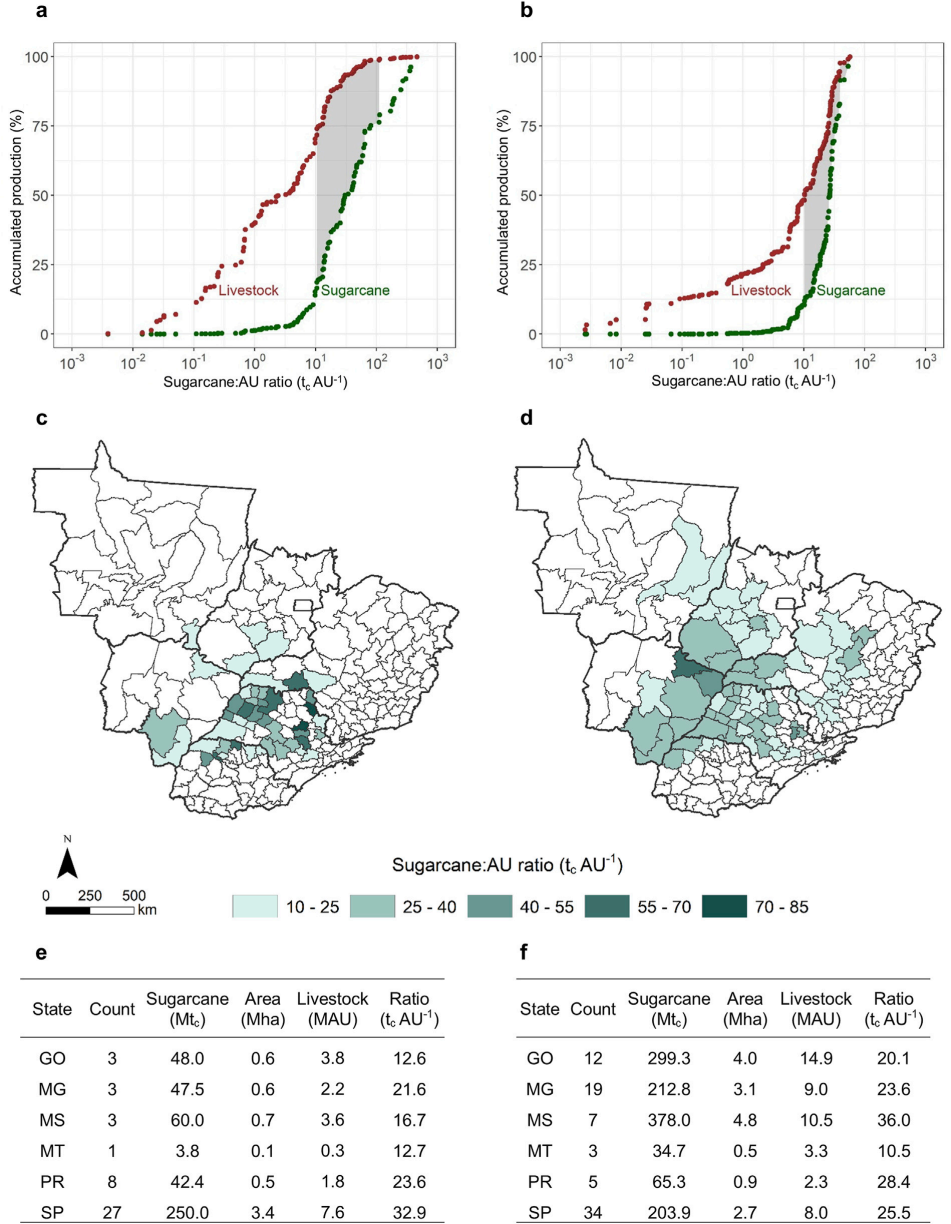
Potential areas for sugarcane-livestock integration through biorefineries. **(a,b)** Aggregated percentages of sugarcane and livestock production sorted by sugarcane-livestock ratio for the current and expansion scenarios. Significant coexistence of both activities is observed for sugarcane-livestock ratios within 10–100 t_c_ AU^−1^ (gray-shaded ranges). **(c,d)** Spatial distribution of geographic microregions with sugarcane-livestock ratios within 10–100 t_c_ AU^−1^ for the current and expansion scenarios. **(e,f)** Production data aggregated at the state level for the current and expansion scenarios.

Sorting the microregions by the sugarcane:AU ratio shows an exciting pattern. In the current sugarcane scenario (Figure 1a), both activities coexist within a range of approximately 10–100 t_c_ AU^−1^. This range is approximately maintained in the expansion scenario (Figure 1b), although it shifts to the lower boundary. Areas below 10 t_c_ AU^−1^ have significant livestock production but minimal sugarcane. Conversely, areas above 100 t_c_ AU^−1^ exhibit significant sugarcane production but limited livestock. The coexistence range (10–100 t_c_ AU^−1^) is found in 45 microregions of the current scenario (Figure 1c) and 80 microregions of the expansion scenario (Figure 1d). These areas of the current scenario produce 451.7 Mt_c_ with 19.3 MAU of livestock (Figure 1e). Meanwhile, the areas of the expansion scenario have the potential to produce 1194.0 Mt_c_ with 48.0 MAU of livestock (Figure 1e). These numbers underscore the potential of technologies that convert sugarcane biomass into livestock feed.

Since the expansion of crops such as sugarcane has raised the debate of direct and indirect land use change impacts, a conservative sugarcane expansion scenario is proposed to occur over pasturelands, as recently occurred (Hernandes et al., 2022). Current livestock is primarily produced extensively on pastureland, and shifting to feedlots is part of the proposed strategy of intensification for sugarcane expansion (Rinke Dias de Souza et al., 2021). Moreover, biorefined feed is one possible tool to be integrated in strategies for intensification without livestock displacement.

## 3 Economies of scale and multiple streams in biorefineries

**FIGURE 2:**
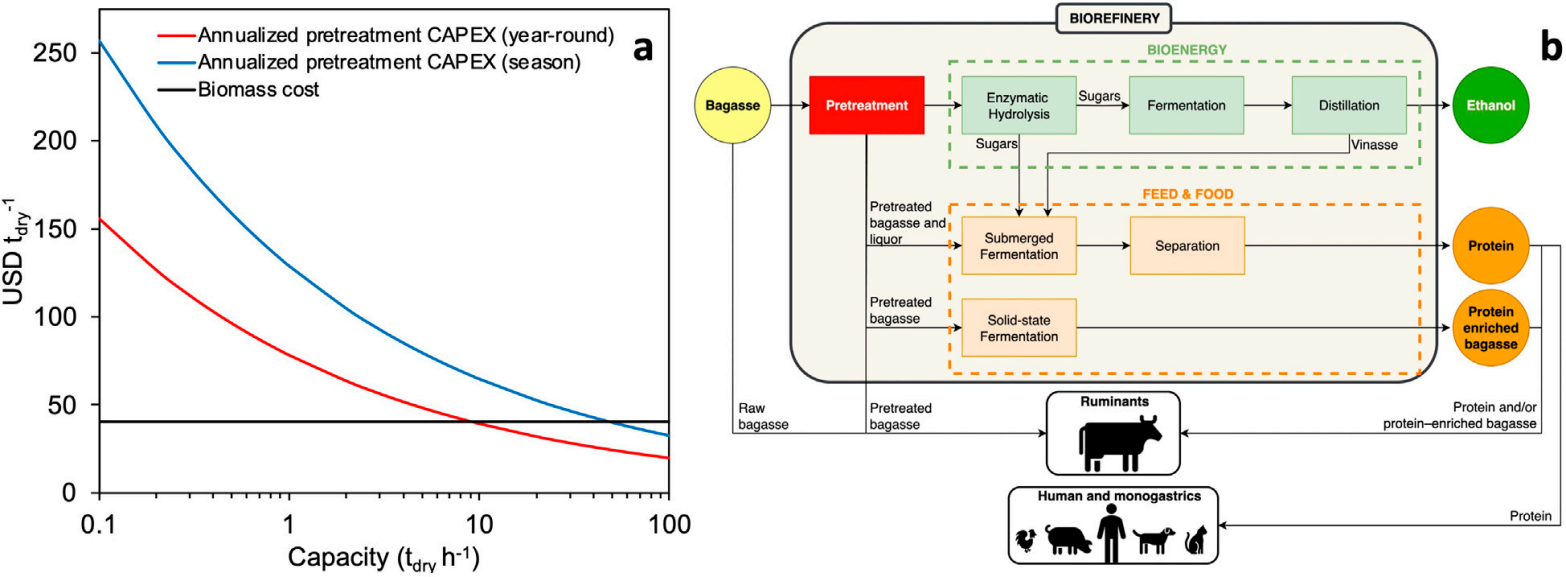
Potential benefits of fuel-feed-food integration due to economies of scale and multiple streams in biorefineries. **(a)** Comparison of a representative lignocellulose cost (40 USD/dry tonne) and the annualized CAPEX of a biorefinery’s pretreatment area (steam-explosion technology). The annualized CAPEX is reported per dry tonne of processed biomass and considers seasonal (200 days) and year-round (330 days) operations. **(b)** Simplified flowchart illustrating biorefinery streams that may be used as feed and food.

Biomass processing costs benefit from economies of scale in biorefineries. Figure 2A compares a representative lignocellulose cost (40 USD/t_dry_) with the annualized CAPEX of a cellulosic ethanol plant’s pretreatment area using high-temperature aqueous acidic pretreatments, like steam explosion. The graph shows CAPEX in USD/t_dry_ for seasonal (200 days, approximately April−November) and year-round (330 days, complemented by maintenance time) operations. It uses techno-economic data (CAPEX of the pretreatment area, plant capacity, 25-year project lifespan, 12% internal rate of return, short-term scenario) from Junqueira et al. (2017), assuming a scaling exponent of 0.7 (Humbird et al., 2011). Current scales of sugarcane cellulosic ethanol facilities (about 80 ML yr^-1^) correspond to capacities of about 40–50 t_dry_ h^-1^. At these scales, processing costs may be lower than the costs of lignocellulosic feedstocks. However, Figure 2a simplifies the benefits of scale, not considering additional advantages in reactor and control technologies or operational and administrative costs.

Besides economies of scale and year-round operation, lignocellulose biorefineries deal with multiple process streams. Figure 2b shows a simplified process flowchart of lignocellulose biorefining into ethanol and potential feed and food coproducts. Pretreated biomass can be used as feed, as further discussed in Section 4 and Section 5. In the biomass-to-ethanol process, pretreated biomass undergoes enzymatic hydrolysis to convert cellulose and hemicelluloses into monomeric sugars (glucose and xylose), which are then fermented, followed by distillation to obtain ethanol (Su et al., 2020; Raj et al., 2022).

Sugar-rich hydrolysates can be fermented into ethanol or used to produce microbial protein for various diets, including those of monogastric animals (*e.g.*, pork, poultry, and fish), pets (*e.g.*, cats and dogs) and humans (Matassa et al., 2016; Cedeno et al., 2025), as further discussed in Section 6. However, the sugar hydrolysate stream is valuable, and lower-value streams such as vinasse (the aqueous residue from distillation) and pretreatment liquors (*e.g.*, alkaline liquor) may be preferred for microbial growth. Utilizing dilute, low-value streams would not compete with sugar fermentation but instead with anaerobic digestion to produce biogas (Moraes et al., 2015).

Comparing biomass availability and livestock feed demand is important. Equation 1 represents a hypothetical balance between lignocellulose-derived feed supply and demand,

$${L.s}_{refined}.s_{feed}=N_{AU}.In.s_{feedlot}.s_{intake}.$$ (1)

In Equation 1, feed supply is the product of the lignocellulose availability *L* (t_dry_), the share of lignocellulose that is biorefined *s*_*refined*_ (%), and the share of biorefined lignocellulose that is directed for feed *s*_*feed*_ (%). The feed demand is the product of the livestock population *N*_*AU*_ (AU), the feed intake *In* (t_dry_ AU^−1^), the share of livestock in feedlots with biorefined feed *s*_*feedlot*_ (%), and the share of the intake made of biorefined feed *s*_*intake*_ (%). One t_c_ comprises about 0.13 t_dry_ of stalk fibers (bagasse) and about 0.07 t_dry_ of straw, although straw availability depends on local conditions (Negrão et al., 2021). Lignocellulose availability thus becomes 0.20 t_dry_/t_c_. Sugarcane-livestock coexistence ratio (10–100 t_c_ AU^−1^, Figure 1) means *L/N*_*AU*_ of 2–20 t_dry_ AU^−1^. Demand for feedlot ration would occur primarily during the dry season when pasture is scarce, coinciding with the sugarcane harvest season. With a 200-day season and a daily feed intake of 0.01 t_dry_ AU^−1^ (about 2% of the animal’s weight), estimated *In* is about two t_dry_ AU^−1^. After having these estimates for *L/N*_*AU*_ and *In* in Equation 1, matching supply and demand of lignocellulose-derived feed can be adjusted by multiple factors (*s*_*refined*_, *s*_*feed*_, *s*_*feedlot*_, and *s*_*intake*_). Due to this flexibility, livestock feed coproduction in lignocellulose biorefineries must be understood as a versatile concept adaptable to different technologies and strategies.

## 4 Biomass pretreatments

Biomass pretreatments aim to reduce the natural recalcitrance of lignocellulosic biomass. Amongst the many biomass pretreatment technologies (Sun et al., 2016; Chen et al., 2017), this section focuses on three technologies—steam explosion (StEx), mild alkali (MA), and ammonia fiber expansion (AFEX) — that present higher technological readiness levels (TRL) and a recognized potential for dual use (fuel and feed). Table 1 shows feed analysis of raw bagasse and bagasse pretreated by StEx, MA, and AFEX technologies. Digestibility increases from 7.8% in raw bagasse to 47.4%–59.0% after pretreatments, demonstrating the reduced biomass recalcitrance that serves both the conversion into ethanol and the value as feed.

**TABLE 1:** Examples of results from feed analysis of raw bagasse compared to bagasse pretreated by steam explosion (StEx), mild alkaline (MA), and ammonia fiber expansion (AFEX).

Parameters	Raw	StEx*	MA*	AFEX**
Dry matter (%)	85.7	35.0	44.2	94.7
Crude protein (%)^†^	3.0	3.9	3,8	13,8
Neutral detergent fiber (%)	93.5	60.3	72.7	78.0
Acid detergent fiber (%)	78.9	54.1	62.2	61.8
Digestibility (%)	7.8	47.4	56.8	59.0
Total nitrogen (%)	0.48**	0.62**	0.6	2.2

†Total nitrogen × 6.25.

*(Molina et al., 1983).

**(Mokomele et al., 2018b).

Table 2 shows compositional data for raw and pretreated bagasse. The severity of pretreatment reactions affects biomass response, so the data should be seen as examples of each pretreatment type.

**TABLE 2:** Chemical composition of raw bagasse and bagasse pretreated by steam explosion (StEx), mild alkali (MA), and ammonia fiber expansion (AFEX). Data is expressed as percentage of raw bagasse and discriminates solid and liquid streams after StEx and MA pretreatments.

Components	Raw	StEx		MA		AFEX*
	%	Solid %	Liquid %	Solid %	Liquid %	(%)
*Pretreatment yield (%)*	—	*73.6*	*26.4*	*86.0*	*14.0*	—
Cellulose	40.5	39.8	—	42.7	—	39.5
*G-OS*	—	—	1.9	—	0.3	—
*Glucose*	—	—	0.03	—	0.0	—
Xylan	23.1	7.8	—	22.5	—	25.2
*X-OS*	—	—	14.4	—	1.2	—
*Xylose*	—	—	1.3	—	0.0	—
Arabinan	1.6	0.4	—	2.5	—	N.R.
*Arabinose*	—	—	0.7	—	0.0	—
Acetyl groups	3.3	1.0	1.3	0.03	—	N.R.
*Free acetic acid*	—	—	0.2	—	3.1	
Lignin	25.1	21.6	5.8	15.7	8.2	15.9
Ashes	3.0	1.2	—	1.2	—	2.8

Raw bagasse has an extractives content of 3.79% ± 0.48%.

OS, oligosaccharides.

N.R., not reported.

*(Mokomele et al., 2022).

The StEx pretreatment belongs to a family of high-temperature (170–210°C) aqueous acidic pretreatments currently utilized in the pioneer cellulosic ethanol biorefineries (TRL 8–9) (Nascimento et al., 2024). This group of pretreatments includes variations with and without the addition of mineral acids (*e.g.*, H_2_SO_4_), but only the versions without acid addition produce material suitable for livestock feed (Manzano et al., 2000). Batch reactors known as “hydrolyzers” have been used in several sugarcane biorefineries to create “hydrolyzed bagasse” for use as feed (as further discussed in Section 5). As a feed component, StEx improves the bagasse digestibility (Table 1), but loses fiber integrity. From a compositional standpoint (Table 2), lignin and cellulose are mostly preserved in the solid fraction, albeit with structural changes (Langan et al., 2014; Driemeier et al., 2015; 2016; Ruiz et al., 2020). Minor portions are solubilized as oligomers (lignin fragments and gluco-oligosaccharides). Conversely, most xylan is solubilized, predominantly forming xylo-oligosaccharides. Acetyl groups from hemicelluloses are partly released as free acetic acid, while some remain bound to the xylo-oligosaccharides. The release of acetic acid in the aqueous phase is primarily responsible for the low pH of StEx-pretreated bagasse.

Mild alkaline (MA) pretreatments also improve digestibility (Table 1) and bagasse intake (Molina et al., 1983; Ezequiel et al., 2005). Versions of MA pretreatments have been developed and scaled up (TRL 6-7) to be the backbones of cellulosic ethanol biorefineries, such as the Deacetylation and Mechanical Refining (DMR) pathway (Chen et al., 2016; 2019; Li et al., 2021). This technology has been tested with bagasse at low temperatures (55°C–90°C) with NaOH concentration within 0.1%–0.7% for 1–5 h and shows promise for ethanol production and alkaline liquor biodigestion (Lima et al., 2018; Volpi et al., 2021).

Ammonia as a pretreatment reagent has also sparked interest because of the potential for ammonia recovery and the dual use as a nitrogenous nutrient (Zhao et al., 2020). AFEX, an ammonia-based pretreatment, has reached pilot/demonstration scale (TRL 6–7) and is considered for ethanol and feed production (Mokomele et al., 2018a). AFEX is a dry-to-dry process (i.e., no aqueous stream) that treats lignocellulosic biomass with ammonia at moderate temperatures (100°C–140°C) and pressures (7–28 bar), during 15–60 min, followed by rapid decompression. The process envisions decentralized facilities (depots) near the fields (Jin and Dale, 2019; Mokomele et al., 2022). AFEX-treated bagasse significantly increases total nitrogen content (Table 1) (Mokomele et al., 2018b), primarily due to non-protein nitrogen linked during pretreatment. This provides valuable nitrogen for ruminants, but raises concerns about acetamide formation, which has been detected in milk and beef from AFEX-fed cattle, warranting further regulatory and health investigations (Bals et al., 2019). Notably, the rumen microbial population can convert non-protein nitrogen to microbial protein, turning a low-cost nitrogen source into an important protein supply for the animal (Huntington and Archibeque, 2000).

## 5 Rebalancing ruminant diets

Livestock diets must be tailored for production goals, considering local constraints and the producers’ objectives. Among possible scenarios for livestock production, scarcity justifies the utilization of low-digestibility feedstuff such as raw bagasse. However, it results in low dry-matter intake, sometimes merely to alleviate hunger and keep the animals alive.

Conversely, beef feedlot diets in Brazil have shifted towards high-concentrate feed, *i.e.*, low-fiber, high-energy, high-protein feedstuff (Millen et al., 2009; Oliveira and Millen, 2014; Pinto and Millen, 2019). Advantages of high-concentrate diets include lower operational costs, better feed efficiency, and a higher rate of fat deposition, enabling precocious finishing and slaughtering to produce superior-quality meat. The inclusion of fibrous feed is thus trending towards the minimum for proper rumen function. Raw bagasse is inserted in amounts as low as 9%–14% of dry matter (Bulle et al., 2002), being a good source of fiber, especially if nearby a biorefinery, also saving land area that would otherwise be needed to produce an equivalent amount of fiber, most likely corn silage (Silvestre and Millen, 2021). A serious drawback of high-concentrate diets is the competition for edible resources, undermining the unique advantage of ruminants in utilizing non-edible fibrous feed, the main reason livestock uses only 14% of human-edible grains (Mottet et al., 2017). High-concentrate diets are also vulnerable to the price volatility of the concentrate, which can be further aggravated by climate change and the growing global demand for food and feed.

Biorefined feed offers a middle ground between the scarcity and the high-concentrate diets. It provides better digestibility than crude fiber (Table 1) but is less nutritious than concentrates. Since the 1980s, several attempts have been made to increase bagasse digestibility and inclusion in ruminant diets (Burgi, 1985; Lanna et al., 1999; Manzano et al., 2000). Steam treatment of bagasse (StEx) became the preferred choice before the development of cellulosic ethanol biorefineries. StEx bagasse has been included up to 40%–60% (dry matter) in ruminant diets. However, the StEx process generates organic acids, presents an intrinsic low pH, and destroys fiber integrity (Burgi, 1985; Medeiros and Machado, 1993). The lower fiber effectiveness in stimulating rumination induces a more acidic ruminal environment, adding stress to the ruminal buffering system, as demonstrated by Weiss et al. (2017), who further showed that increasing particle length can mitigate the issue. Alkaline pretreatments that preserve fiber effectiveness and help to control the ruminal pH might be of interest if their enhanced digestibility allows higher inclusions in competitive diets. Nevertheless, the low protein content in raw and pretreated bagasse (Table 1) is a key limitation for balancing a diet enriched in biorefined feed. Addressing the low protein content in bagasse through protein production in lignocellulose biorefineries could help balance such diets, offering a valuable adaptation strategy for regions facing climate change-induced challenges like droughts (Han and Singh, 2023).

## 6 Microbial protein

Microbial protein, also known as single-cell protein (SCP), refers to dried cells of microorganisms used as functional ingredients in animal feed and human food (Banks et al., 2022; Li et al., 2024; Cedeno et al., 2025). Animal feed or human food applications require compliance with specific safety and regulatory frameworks. Feed-grade microbial protein typically undergoes less stringent purification than food-grade products. However, it must still meet regulatory standards for safety, toxicity, and nutritional adequacy before market approval (Grigore et al., 2025). Human food applications demand more rigorous safety evaluations, higher purity levels, and regulatory approvals (Lähteenmäki-Uutela et al., 2021). Various species of microorganisms, including fungi, bacteria, and algae, are used to produce microbial protein, with many of them being Generally Recognized as Safe (GRAS) and having a Protein Digestibility-Corrected Amino Acid Score (PDCAAS) close to 1 (Koukoumaki et al., 2024). It has been proven to be highly nutritious, presenting excellent amino acid profiles, particularly highlighting lysine, methionine, and threonine, making it ideal as a supplement in animal diets (Sharif et al., 2021). Also, it offers an alternative to plant-based protein with advantages such as higher protein content, faster growth and production, and independence of seasonal variations (Tian et al., 2023). Beyond nutritional benefits, microbial protein production offers potential environmental advantages, with life cycle assessments indicating significantly lower GHG emissions compared to soybean meal or fishmeal (Matassa et al., 2016).

Microbial protein can be produced by either solid-state or submerged fermentation, followed by downstream processing of microbial biomass. Solid-state fermentation cultivates microorganisms on solid substrates without free-flowing water, mimicking natural habitats and allowing efficient substrate utilization (Soccol et al., 2017). Pretreated bagasse and straw are suitable substrates containing carbohydrates accessible for microbial growth. For example, assays at laboratory scale using *Pleurotus ostreatus* and *Fusarium venenatum* have reached 25 and 33 g of microbial protein per kg of treated bagasse (Lourens et al., 2025).

In submerged fermentation, microorganisms are cultivated in liquid media, which allows for better control of environmental conditions, nutrient availability, and scalability of the process. The sugar-rich enzymatic hydrolysate (Figure 2B) may be used for microbial protein production. This method allows a higher yield of microbial protein, compared to solid-state fermentation, reaching levels of 100–300 g per kg of substrate (Zhao et al., 2023; Sun et al., 2024). However, using sugar hydrolysate for microbial protein competes with ethanol production. An alternative is to use lower-value streams from biorefining, such as pretreatment liquor or vinasse, which can be environmentally hazardous if not treated adequately, and may require conditioning (*e.g.*, pH adjustment, detoxification) before microbial cultivation. The superior process control of submerged fermentation may enable enhanced-quality microbial products, possibly for higher-value applications, including monogastric feed and even human food (Cedeno et al., 2025).

## 7 Concluding remarks

This Perspective explored the potential of integrating feed and food coproducts within lignocellulose biorefineries. It showed that the sugarcane industry is particularly suited for this due to its leadership in cellulosic ethanol and proximity to livestock production in Brazil’s Center-South region. Lignocellulose-derived feed and food is presented as a broad concept that encompasses multiple technologies, including the dual use of pretreated biomass (fuel and feed), the production of microbial protein, and the rebalancing of livestock diets. Advancing R&D is crucial as the cellulosic ethanol industry is new, still needing reductions in biomass processing costs and gains in product revenues to enhance its sustainability.

The breadth of the topic covered in this Perspective indicates that lignocellulose biorefining might open a promising new domain in feed and food science and technology, encompassing sustainability, engineering, nutrition, health, safety, and regulatory issues. Although there is potential for incremental additions to the existing lignocellulose biorefining technologies, this path may lead to a limited impact. A broader and more impactful approach requires the concerted action of policy, investment, and R&D to transform how lignocellulose biorefining is thought, designed, and deployed.

## Data Availability

The original contributions presented in the study are included in the article/supplementary material, further inquiries can be directed to the corresponding author.

## References

[B1] Bals B. Teymouri F. Haddad D. Julian W. A. Vismeh R. Jones A. D. (2019). Presence of acetamide in milk and beef from cattle consuming AFEX-treated crop residues. J. Agric. Food Chem. 67, 10756–10763. 10.1021/acs.jafc.9b04030 31483626 PMC6764021

[B2] Banks M. Johnson R. Giver L. Bryant G. Guo M. (2022). Industrial production of microbial protein products. Curr. Opin. Biotechnol. 75, 102707. 10.1016/j.copbio.2022.102707 35276510

[B3] Bulle M. L. de M. Ribeiro F. G. Leme P. R. Titto E. A. L. Lanna D. P. D. (2002). Desempenho de Tourinhos Cruzados em Dietas de Alto Teor de Concentrado com Bagaço de Cana-de-Açúcar como Único Volumoso. Rev. Bras. Zootec. 31, 444–450. 10.1590/S1516-35982002000200020

[B4] Burgi R. (1985). “Produção do bagaço de cana-de-açúcar (Saccharum sp L.) auto-hidrolisado e avaliação para ruminantes,” Piracicaba: University of São Paulo.

[B5] Cedeno F. R. P. Olubiyo O. J. Ferreira S. (2025). From microbial proteins to cultivated meat for alternative meat-like products: a review on sustainable fermentation approaches. J. Biol. Eng. 19, 44. 10.1186/s13036-025-00509-9 40369620 PMC12077041

[B6] Chen X. Wang W. Ciesielski P. Trass O. Park S. Tao L. (2016). Improving sugar yields and reducing enzyme Loadings in the deacetylation and mechanical refining (DMR) process through multistage disk and szego refining and corresponding techno-economic analysis. ACS Sustain Chem. Eng. 4, 324–333. 10.1021/acssuschemeng.5b01242

[B7] Chen H. Liu J. Chang X. Chen D. Xue Y. Liu P. (2017). A review on the pretreatment of lignocellulose for high-value chemicals. Fuel Process. Technol. 160, 196–206. 10.1016/j.fuproc.2016.12.007

[B8] Chen X. Crawford N. Wang W. Kuhn E. Sievers D. Tao L. (2019). Kinetics and Rheological Behavior of higher solid (solids >20%) enzymatic hydrolysis reactions using dilute acid pretreated, deacetylation and disk refined, and deacetylation and Mechanical refined (DMR) corn stover Slurries. ACS Sustain Chem. Eng. 7, 1633–1641. 10.1021/acssuschemeng.8b05391

[B9] CONAB (2023). Acompanhamento da Safra Brasileira de Cana-de-açúcar. Safra 2023/24. Bras.

[B10] de Souza Dias M. O. Maciel Filho R. Mantelatto P. E. Cavalett O. Rossell C. E. V. Bonomi A. (2015). Sugarcane processing for ethanol and sugar in Brazil. Environ. Dev. 15, 35–51. 10.1016/j.envdev.2015.03.004

[B11] Dees J. P. Sagues W. J. Woods E. Goldstein H. M. Simon A. J. Sanchez D. L. (2023). Leveraging the bioeconomy for carbon drawdown. Green Chem. 25, 2930–2957. 10.1039/d2gc02483g

[B12] Driemeier C. Mendes F. M. Santucci B. S. Pimenta M. T. B. (2015). Cellulose co-crystallization and related phenomena occurring in hydrothermal treatment of sugarcane bagasse. Cellulose 22, 2183–2195. 10.1007/s10570-015-0638-7

[B13] Driemeier C. Oliveira M. M. Curvelo A. A. S. (2016). Lignin contributions to the nanoscale porosity of raw and treated lignocelluloses as observed by calorimetric thermoporometry. Ind. Crops Prod. 82, 114–117. 10.1016/j.indcrop.2015.11.084

[B14] Eckert C. T. Frigo E. P. Albrecht L. P. Albrecht A. J. P. Christ D. Santos W. G. (2018). Maize ethanol production in Brazil: characteristics and perspectives. Renew. Sustain. Energy Rev. 82, 3907–3912. 10.1016/j.rser.2017.10.082

[B15] Ezequiel J. M. B. Queiroz M. A. Á. Galati R. L. Mendes A. R. Pereira E. M. de O. Faturi C. (2005). Processamento da cana-de-açúcar: efeitos sobre a digestibilidade, o consumo e a taxa de passagem. Rev. Bras. Zootec. 34, 1704–1710. 10.1590/S1516-35982005000500032

[B16] Grigore D.-M. Mircea M.-L. Pogurschi E. N. (2025). Toward sustainable Broiler production: Evaluating microbial protein as Supplementation for Conventional feed proteins. Agriculture 15, 1486. 10.3390/agriculture15141486

[B17] Han J. Singh V. P. (2023). A review of widely used drought indices and the challenges of drought assessment under climate change. Environ. Monit. Assess. 195, 1438. 10.1007/s10661-023-12062-3 37943470

[B18] Hernandes T. A. D. Duft D. G. Luciano A. C. dos S. Leal M. R. L. V. Cavalett O. (2021). Identifying suitable areas for expanding sugarcane ethanol production in Brazil under conservation of environmentally relevant habitats. J. Clean. Prod. 292, 125318. 10.1016/j.jclepro.2020.125318

[B19] Hernandes T. A. D. de Oliveira Bordonal R. Duft D. G. Leal M. R. L. V. (2022). Implications of regional agricultural land use dynamics and deforestation associated with sugarcane expansion for soil carbon stocks in Brazil. Reg. Environ. Change 22, 49. 10.1007/s10113-022-01907-1

[B20] Humbird D. Davis R. Tao L. Kinchin C. Hsu D. Aden A. (2011). “Process Design and economics for biochemical conversion of lignocellulosic biomass to ethanol: dilute-acid pretreatment and enzymatic hydrolysis of corn stover,”. Golden, CO (United States). 10.2172/1013269

[B21] Huntington G. B. Archibeque S. L. (2000). Practical aspects of urea and ammonia metabolism in ruminants. J. Anim. Sci. 77, 1. 10.2527/jas2000.77E-Suppl1y

[B22] IBGE (2022). PPM - Pesquisa da Pecuária Municipal. Available online at: https://sidra.ibge.gov.br/pesquisa/ppm/tabelas (Accessed November 29, 2022).

[B23] Jin M. Dale B. E. (2019). “AFEX^TM^ pretreatment-based biorefinery technologies,” in Handbook of biorefinery research and technology (Dordrecht: Springer Netherlands), 1–16. 10.1007/978-94-007-6724-9_2-2

[B24] Junqueira T. L. Cavalett O. Bonomi A. (2016). The virtual sugarcane biorefinery—a Simulation tool to support public Policies Formulation in bioenergy. Ind. Biotechnol. 12, 62–67. 10.1089/ind.2015.0015

[B25] Junqueira T. L. Chagas M. F. Gouveia V. L. R. Rezende M. C. A. F. Watanabe M. D. B. Jesus C. D. F. (2017). Techno-economic analysis and climate change impacts of sugarcane biorefineries considering different time horizons. Biotechnol. Biofuels 10, 50. 10.1186/s13068-017-0722-3 28293288 PMC5348788

[B26] Klein B. C. de Mesquita Sampaio I. L. Mantelatto P. E. Filho R. M. Bonomi A. (2019). Beyond ethanol, sugar, and electricity: a critical review of product diversification in Brazilian sugarcane mills. Biofuels, Bioprod. Biorefining 13, 809–821. 10.1002/bbb.1969

[B27] Koukoumaki D. I. Tsouko E. Papanikolaou S. Ioannou Z. Diamantopoulou P. Sarris D. (2024). Recent advances in the production of single cell protein from renewable resources and applications. Carbon Resour. Convers. 7, 100195. 10.1016/j.crcon.2023.07.004

[B28] Kumar B. Bhardwaj N. Agrawal K. Chaturvedi V. Verma P. (2020). Current perspective on pretreatment technologies using lignocellulosic biomass: an emerging biorefinery concept. Fuel Process. Technol. 199, 106244. 10.1016/j.fuproc.2019.106244

[B29] Lähteenmäki-Uutela A. Rahikainen M. Lonkila A. Yang B. (2021). Alternative proteins and EU food law. Food control. 130, 108336. 10.1016/j.foodcont.2021.108336

[B30] Langan P. Petridis L. O’Neill H. M. Pingali S. V. Foston M. Nishiyama Y. (2014). Common processes drive the thermochemical pretreatment of lignocellulosic biomass. Green Chem. 16, 63–68. 10.1039/c3gc41962b

[B31] Langholtz M. H. (2024). 2023 Billion-Ton Report: an assessment of U.S. Renewable carbon resources. Oak Ridge.

[B32] Lanna D. P. D. Morais J. P. Boin C. Fox D. G. Leme P. R. Castro F. B. de (1999). Desempenho e composição corporal de novilhas alimentadas com dois níveis de concentrado e bagaço de cana submetidos a diferentes processos de hidrólise. Rev. Bras. Zootec. 28, 412–420. 10.1590/S1516-35981999000200027

[B33] Li Y. Chen X. Sievers D. A. (2021). Modelling a compressible packed bed flow-through washing and deacetylation reactor for corn stover pretreatment. Chem. Eng. J. 415, 128918. 10.1016/j.cej.2021.128918

[B34] Li Y. P. Ahmadi F. Kariman K. Lackner M. (2024). Recent advances and challenges in single cell protein (SCP) technologies for food and feed production. NPJ Sci. Food 8, 66. 10.1038/s41538-024-00299-2 39294139 PMC11410949

[B35] Lima C. S. Rabelo S. C. Ciesielski P. N. Roberto I. C. Rocha G. J. M. Driemeier C. (2018). Multiscale Alterations in sugar cane bagasse and straw Submitted to alkaline deacetylation. ACS Sustain Chem. Eng. 6, 3796–3804. 10.1021/acssuschemeng.7b04158

[B36] Lourens V. Bosman C. E. Petersen A. M. Coetzee G. Görgens J. F. van Rensburg E. (2025). Simultaneous enzymatic hydrolysis and bioconversion of deacetylated and disk refined sugarcane bagasse to single-cell protein: an experimental investigation and techno-economic analysis. Biochem. Eng. J. 218, 109691. 10.1016/j.bej.2025.109691

[B37] Manzano R. P. Fukushima R. S. Gomes J. D. F. Garippo G. (2000). Digestibilidade do bagaço de cana-de-açúcar tratado com reagentes químicos e pressão de vapor. Rev. Bras. Zootec. 29, 1196–1204. 10.1590/S1516-35982000000400034

[B38] Manzatto C. V. Assad E. D. Bacca J. F. M. Zaroni M. J. Pereira S. E. M. (2009). Zoneamento agroecológico da cana-de-açúcar: expandir a produção, preservar a vida, garantir o futuro. Brasilia.

[B39] Matassa S. Boon N. Pikaar I. Verstraete W. (2016). Microbial protein: future sustainable food supply route with low environmental footprint. Microb. Biotechnol. 9, 568–575. 10.1111/1751-7915.12369 27389856 PMC4993174

[B40] Medeiros S. R. Machado P. F. (1993). Effect of the Replacement of steam treated Sugarne bagasse by Milo upon performance of beef cattle. Cali: Livestock Research for Rural Development, 25–30.

[B41] Menezes F. F. Nascimento V. M. Gomes G. R. Rocha G. J. M. Strauss M. Junqueira T. L. (2023). Depolymerization of enzymatic hydrolysis lignin: review of technologies and opportunities for research. Fuel 342, 127796. 10.1016/j.fuel.2023.127796

[B42] Millen D. D. Pacheco R. D. L. Arrigoni M. D. B. Galyean M. L. Vasconcelos J. T. (2009). A snapshot of management practices and nutritional recommendations used by feedlot nutritionists in Brazil. J. Anim. Sci. 87, 3427–3439. 10.2527/jas.2009-1880 19574564

[B43] Mokomele T. da Costa Sousa L. Balan V. Rensburg E. V. Dale B. E. Gorgens J. F. (2018a). Ethanol production potential from AFEXTM and steam exploded sugarcane residues for sugarcane biorefineries. Biotechnol. Biofuels, 1–21. 10.1186/s13068-018-1130-z 29755586 PMC5934847

[B44] Mokomele T. da Costa Sousa L. Bals B. Balan V. Goosen N. Dale B. E. (2018b). Using steam explosion or AFEX^TM^ to produce animal feeds and biofuel feedstocks in a biorefinery based on sugarcane residues. Biofuels, Bioprod. Biorefining 12, 978–996. 10.1002/bbb.1927

[B45] Mokomele T. da Costa Sousa L. Colbert A. Dale B. E. Görgens J. F. Balan V. (2022). Coupling AFEX and steam-exploded sugarcane residue pellets with a room temperature CIIII-activation step lowered enzyme dosage requirements for sugar conversion. Chem. Eng. J. 446, 137117. 10.1016/j.cej.2022.137117

[B46] Molina E. Boza J. Aguilera J. F. (1983). Nutritive value for ruminants of sugar cane bagasse ensiled after spray treatment with different levels of NaOH. Anim. Feed Sci. Technol. 9, 1–17. 10.1016/0377-8401(83)90074-3

[B47] Moraes B. S. Zaiat M. Bonomi A. (2015). Anaerobic digestion of vinasse from sugarcane ethanol production in Brazil: challenges and perspectives. Renew. Sustain. Energy Rev. 44, 888–903. 10.1016/j.rser.2015.01.023

[B48] Mottet A. de Haan C. Falcucci A. Tempio G. Opio C. Gerber P. (2017). Livestock: on our plates or eating at our table? A new analysis of the feed/food debate. Glob. Food Sec 14, 1–8. 10.1016/j.gfs.2017.01.001

[B49] Muscat A. de Olde E. M. de Boer I. J. M. Ripoll-Bosch R. (2020). The battle for biomass: a systematic review of food-feed-fuel competition. Glob. Food Sec 25, 100330. 10.1016/j.gfs.2019.100330

[B50] Nascimento V. M. Nakanishi S. C. de Oliveira Filho C. A. da Conceição Gomes A. de Castro A. M. Torres A. P. R. (2024). Differentiating bagasse and straw as feedstocks for sugarcane cellulosic ethanol: Insights from pilot-scale pretreatments. Bioenergy Res. 17, 1533–1542. 10.1007/s12155-024-10751-6

[B51] Negrão D. R. Grandis A. Buckeridge M. S. Rocha G. J. M. Leal M. R. L. V. Driemeier C. (2021). Inorganics in sugarcane bagasse and straw and their impacts for bioenergy and biorefining: a review. Renew. Sustain. Energy Rev. 148, 111268. 10.1016/j.rser.2021.111268

[B52] Oliveira C. A. Millen D. D. (2014). Survey of the nutritional recommendations and management practices adopted by feedlot cattle nutritionists in Brazil. Anim. Feed Sci. Technol. 197, 64–75. 10.1016/j.anifeedsci.2014.08.010 PMC12175091 40534783

[B53] Paone E. Tabanelli T. Mauriello F. (2020). The rise of lignin biorefinery. Curr. Opin. Green Sustain Chem. 24, 1–6. 10.1016/j.cogsc.2019.11.004

[B73] Petrielli G. P. Nogueira G. P. Henzler D. de S. de Souza N. R. D. Bruno K. M. B. Luciano A. C. dos S. (2023). Integrating carbon footprint to spatialized modeling: The mitigation potential of sugarcane ethanol production in the Brazilian Center-South. Resour. Conserv. Recycl. 189. 10.1016/j.resconrec.2022.106725

[B54] Pinto A. C. J. Millen D. D. (2019). Nutritional recommendations and management practices adopted by feedlot cattle nutritionists: the 2016 Brazilian survey. Can. J. Anim. Sci. 99, 392–407. 10.1139/cjas-2018-0031

[B55] Raj T. Chandrasekhar K. Naresh Kumar A. Rajesh Banu J. Yoon J.-J. Kant Bhatia S. (2022). Recent advances in commercial biorefineries for lignocellulosic ethanol production: current status, challenges and future perspectives. Bioresour. Technol. 344, 126292. 10.1016/j.biortech.2021.126292 34748984

[B56] Rinke Dias de Souza N. Lopes Junqueira T. Cavalett O. (2021). Opportunities and challenges for bioenergy-livestock integrated systems in Brazil. Ind. Crops Prod. 173, 114091. 10.1016/j.indcrop.2021.114091

[B74] Rudorff B. F. T. de Aguiar D. A. da Silva W. F. Sugawara L. M. Adami M. Moreira M. A. (2010). Studies on the rapid expansion of sugarcane for ethanol production in São Paulo state (Brazil) using Landsat data. Remote Sens. (Basel) 2, 1057–1076. 10.3390/rs2041057

[B57] Ruiz H. A. Conrad M. Sun S. N. Sanchez A. Rocha G. J. M. Romaní A. (2020). Engineering aspects of hydrothermal pretreatment: from batch to continuous operation, scale-up and pilot reactor under biorefinery concept. Bioresour. Technol. 299, 122685. 10.1016/j.biortech.2019.122685 31918970

[B58] Scholey D. V. Burton E. J. Williams P. E. V. (2016). The bio refinery; producing feed and fuel from grain. Food Chem. 197, 937–942. 10.1016/j.foodchem.2015.11.063 26617037

[B59] Sharif M. Zafar M. H. Aqib A. I. Saeed M. Farag M. R. Alagawany M. (2021). Single cell protein: Sources, mechanism of production, nutritional value and its uses in aquaculture nutrition. Aquaculture 531, 735885. 10.1016/j.aquaculture.2020.735885

[B60] Silvestre A. M. Millen D. D. (2021). The 2019 Brazilian survey on nutritional practices provided by feedlot cattle consulting nutritionists. Rev. Bras. Zootec. 50, e20200189. 10.37496/rbz5020200189

[B61] Singh N. Singhania R. R. Nigam P. S. Dong C.Di Patel A. K. Puri M. (2022). Global status of lignocellulosic biorefinery: challenges and perspectives. Bioresour. Technol. 344, 126415. 10.1016/j.biortech.2021.126415 34838977

[B62] Soccol C. R. Costa E. S. F. da Letti L. A. J. Karp S. G. Woiciechowski A. L. Vandenberghe L. P. de S. (2017). Recent developments and innovations in solid state fermentation. Biotechnol. Res. Innovation 1, 52–71. 10.1016/j.biori.2017.01.002

[B63] Su T. Zhao D. Khodadadi M. Len C. (2020). Lignocellulosic biomass for bioethanol: Recent advances, technology trends, and barriers to industrial development. Curr. Opin. Green Sustain Chem. 24, 56–60. 10.1016/j.cogsc.2020.04.005

[B64] Sun S. Sun S. Cao X. Sun R. (2016). The role of pretreatment in improving the enzymatic hydrolysis of lignocellulosic materials. Bioresour. Technol. 199, 49–58. 10.1016/j.biortech.2015.08.061 26321216

[B65] Sun W. Zhang Z. Li X. Lu X. Liu G. Qin Y. (2024). Production of single cell protein from brewer’s spent grain through enzymatic saccharification and fermentation enhanced by ammoniation pretreatment. Bioresour. Technol. 394, 130242. 10.1016/j.biortech.2023.130242 38145760

[B66] Tian Y. Li J. Meng J. Li J. (2023). High-yield production of single-cell protein from starch processing wastewater using co-cultivation of yeasts. Bioresour. Technol. 370, 128527. 10.1016/j.biortech.2022.128527 36572157

[B67] Vandenberghe L. P. S. Valladares-Diestra K. K. Bittencourt G. A. Zevallos Torres L. A. Vieira S. Karp S. G. (2022). Beyond sugar and ethanol: the future of sugarcane biorefineries in Brazil. Renew. Sustain. Energy Rev. 167, 112721. 10.1016/j.rser.2022.112721

[B68] Volpi M. P. C. Fuess L. T. Moraes B. S. (2021). Anaerobic co-digestion of residues in 1G2G sugarcane biorefineries for enhanced electricity and biomethane production. Bioresour. Technol. 330, 124999. 10.1016/j.biortech.2021.124999 33780712

[B69] Weiss C. P. Gentry W. W. Meredith C. M. Meyer B. E. Cole N. A. Tedeschi L. O. (2017). Effects of roughage inclusion and particle size on digestion and ruminal fermentation characteristics of beef steers. J. Anim. Sci. 95, 1707. 10.2527/jas2016.1330 28464079

[B70] Xavier A. C. King C. W. Scanlon B. R. (2016). Daily gridded meteorological variables in Brazil (1980–2013). Int. J. Climatol. 36, 2644–2659. 10.1002/joc.4518

[B71] Zhao C. Shao Q. Chundawat S. P. S. (2020). Recent advances on ammonia-based pretreatments of lignocellulosic biomass. Bioresour. Technol. 298, 122446. 10.1016/j.biortech.2019.122446 31791921

[B72] Zhao S. Wang Z.-B. Wang Y.-C. Yang P.-Y. Luo X.-M. Wu A.-M. (2023). Sustainable coproduction of xylooligosaccharide, single-cell protein and lignin-adsorbent through whole components’ utilization of sugarcane bagasse with high solid loading. Sep. Purif. Technol. 308, 122916. 10.1016/j.seppur.2022.122916

